# Associations between executive functioning, challenging behavior, and quality of life in children and adolescents with and without neurodevelopmental conditions

**DOI:** 10.3389/fpsyg.2022.1022700

**Published:** 2022-10-20

**Authors:** Thomas W. Frazier, Ethan Crowley, Andy Shih, Vijay Vasudevan, Arun Karpur, Mirko Uljarevic, Ru Ying Cai

**Affiliations:** ^1^Department of Psychology, John Carroll University, University Heights, OH, United States; ^2^Science and Public Health Department, Autism Speaks, New York, NY, United States; ^3^The School of Psychological Sciences, University of Melbourne, Melbourne, VIC, Australia; ^4^Aspect Research Centre for Autism Practice, French’s Forest, NSW, Australia

**Keywords:** challenging behavior, developmental disability, executive function, social skills, quality of life, functional impact

## Abstract

The present study sought to clarify the impact of executive and social functioning on challenging behavior and the downstream influence of challenging behavior on quality of life and functioning in a large transdiagnostic sample. Understanding these relationships is crucial for developing and designing tailored intervention strategies. In a cross-sectional study, parent informants of 2,004 children completed measures of executive and social functioning, challenging behavior, child and family quality of life, and reported on functional impacts of challenging behavior. Using structural (path) modeling, analyses evaluated the associations between executive and social functioning, including emotion regulation and risk avoidance, with overall and specific types of challenging behavior. Structural models also examined the influence of challenging behavior on child and family quality of life, including measures of the immediate and extended environment, and functional impacts on the parent/child as well as interactions with the medical/legal systems. Finally, mediational models explored the direct and indirect effects of executive and social functioning on quality of life and impact measures *via* challenging behavior. Results indicated that executive functioning accounts for substantial variance (*R*^2^ = 0.47) in challenging behavior. In turn, challenging behavior accounts for substantial variance in child and family quality of life (*R*^2^ = 0.36) and parent/child impacts (*R*^2^ = 0.31). Exploratory mediational models identified direct effects from executive and social functioning measures on quality of life and functional impacts and indirect effects for executive functioning *via* challenging behavior. These findings support the development of new intervention strategies and suggest the need to measure executive functioning when assessing and tailoring the treatment of challenging behavior in clinical practice.

## Introduction

Challenging behaviors (oftentimes referred to as disruptive behaviors and subsumed under broader externalizing spectra; Lecavalier, 2006; Matson and Nebel-Schwalm, 2007; Hattier et al., 2011; Konst et al., 2013; Murray et al., 2021) are a defining characteristic of oppositional defiant and conduct disorders. However, challenging behaviors are not specific to oppositional defiant and conduct disorders, rather, these behaviors frequently occur across a range of neuropsychiatric and neurodevelopmental disorders, including, but not limited to, autism spectrum disorder (ASD; Lecavalier, 2006) and attention-deficit/hyperactivity disorder (ADHD; Kang and Kwack, 2019). Further, these behaviors are also frequently seen in children with a range of psychiatric conditions, including anxiety (Chung et al., 2019) and mood (Youngstrom et al., 2021) disorders, and in neurotypical children (Tang et al., 2013; Fahmie et al., 2020). In many cases, even when low in frequency, and regardless of the primary diagnosis, challenging behavior can have wide ranging effects, including significant impacts on all aspects of child functioning (Davico et al., 2022), access to the social environment and support (Glenn et al., 2021), caregiver stress (Bradshaw et al., 2021), and quality of life (Markowitz et al., 2016). Yet, little is known about what specific aspects of clinical phenotype and neurobehavioral traits most influence the onset and/or maintenance of challenging behavior.

Executive functioning is the ability to intentionally plan and execute appropriate, adaptive, pro-social behaviors to achieve a goal while simultaneously inhibiting inappropriate behaviors (Diamond, 2013; Moriguchi, 2014). Executive functions have been consistently identified as transdiagnostic correlates of internalizing and externalizing psychopathology in general samples across the life span (Hatoum et al., 2018; Snyder et al., 2019; Friedman et al., 2020; Harden et al., 2020; Wade et al., 2020; Freis et al., 2022). Furthermore, executive functioning deficits occur across a range of neurodevelopmental and neuropsychiatric disorders (McTeague et al., 2016, 2017). Although the majority of disorders are associated with relatively uniform impairments across different aspects of executive functioning and might thus be best characterized by deficits in common executive functioning factor, there are pronounced variations in effect sizes of specific executive functioning deficits in certain conditions (Snyder et al., 2015). For instance, a meta-analysis by Snyder et al. (2015) has suggested more pronounced deficits for updating in ADHD and for responses inhibition in obsessive compulsive disorder. Importantly, these deficits have been identified as transdiagnostic risk factors for challenging behaviors across normative and atypical development, including ASD (Maddox et al., 2018), children with epilepsy (van den Berg et al., 2021), diverse internalizing and externalizing clinical populations (Marleau et al., 2021), and neurotypical children (Romero-Lopez et al., 2017). In normative samples, studies have either found a common executive function factor to be more relevant to externalizing psychopathology rather than specific executive functions (e.g., Friedman et al., 2020) or both common factor and specific executive functions such as shifting-specific and response inhibition to be predictive of externalizing behaviors (Wang et al., 2017; Hatoum et al., 2018). Thus, it is important to further explore whether certain executive functioning subdomains might be more strongly associated with the severity of challenging behaviors across normative and clinical samples. This is particularly important given that impact of executive functioning on challenging behaviors remains unexplored in several neurodevelopmental conditions, including ASD. In addition to executive functioning, emotion regulation has been suggested as a key transdiagnostic process (Aldao et al., 2016; Cludius et al., 2020; Cai et al., 2021). Importantly, in clinical samples, there is emerging evidence that impaired emotion regulation is associated with more severe challenging behaviors across a range of disorders, including ASD (Cai et al., 2018; Beck et al., 2020) and disruptive behavior disorders (Woltering et al., 2016; Wang et al., 2017). However, to the best of our knowledge, no study to date has explored the influence of key executive functioning subdomains and related transdiagnostic processes such as emotion regulation on challenging behaviors in a mixed sample of individuals who are typically developing and with neurodevelopmental conditions. The present study addresses this gap by including a recently developed informant-reported measures designed to comprehensively capture diverse aspects of executive functioning including the sequencing/working memory, response inhibition, and set shifting domains, and other important, related transdiagnostic processes, including emotion regulation and risk avoidance, with the goal of understanding specific relationships between executive functioning domains and specific types of challenging behavior.

Individual differences in social development have also been linked to challenging behavior, irrespective of the primary diagnosis, with stronger development of social skills associated with fewer behavior problems (Hukkelberg et al., 2019). However, the specificity of this broad relationship is unclear as no studies have comprehensively evaluated specific facets of social functions and their relationships with different types of challenging behaviors. While often conceptualized and measured as a unitary construct (Constantino and Todd, 2003; Constantino et al., 2007), in support of more recent models of social functioning (e.g., framework proposed by the Research Domain Criteria) recent studies have demonstrated multifaceted nature of the social functioning domain, identifying a consistent set of more specific social subdomains (Frazier et al., 2014; Uljarevic et al., 2019, 2020), including: social motivation (a general tendency toward social interaction), basic social communication skills (sometimes combining and other times separating verbal and non-verbal skills such as gestures, eye contact, social distance, etc.), affiliation (relationship development and maintenance), and social recognition (identifying intentions, motivations, and feelings in others and using social cues to guide behavior). However, previous research in ASD and other disorders has not clearly delineated which aspects of social functioning have the most important role in the development and maintenance of challenging behaviors. The present study addresses the specificity problem by examining associations between specific social functions and specific types of challenging behaviors.

Studies of young children have suggested a close association between the development of executive and social functions (Alduncin et al., 2014). For example, both cross-sectional and longitudinal studies have reported strong associations between executive functioning and social functioning in ASD (Pellicano, 2007; Freeman et al., 2017). Further, there is emerging evidence of functional inter-dependency (Moriguchi, 2014), with development of executive functions driving age-appropriate social behavior and vice versa. For example, the development of response inhibition may be crucial for the development of reciprocal peer interaction skills, while interactions with parents and peers may be necessary for the development of emotion regulation skills. Thus, as a first step to building a more comprehensive model of challenging behavior, it is important to simultaneously evaluate specific executive and social functioning skills and their influence on challenging behavior.

A comprehensive model of challenging behavior should also include the most important downstream impacts. As stated above, the existing literature suggests that challenging behavior has significant effects on the immediate environment (caregiver stress, reductions in child functioning, family quality of life; Markowitz et al., 2016; Bradshaw et al., 2021; Davico et al., 2022), but can also influence the larger social context (Matson and Nebel-Schwalm, 2007; Glenn et al., 2021). To facilitate a broad view of impacts, the present study collected measures that evaluate key levels of Bronfenbrenner’s ecological systems model (Bronfenbrenner, 1989). This model identifies different levels of relationships between the child and their environment. For the present study, the levels considered were the child’s immediate environment (microsystem), connections between the immediate environment and the indirect environment (mesosystem), and the indirect environment (exosystem). Specifically, to evaluate micro-and mesosystem levels, the study captured child, caregiver, and family quality of life and impacts of challenging behavior on parent employment and child school and social engagement. To evaluate the larger exosystem, parent-reports of financial and external support aspects of quality of life as well as medical and legal interactions were collected (see Supplementary material 1 for the study schema).

### The present study

The first aim of this study was to evaluate executive and social functioning domains as predictors of challenging behavior. Given that executive and social functioning domains represent neurobehavioral traits that develop early (Anderson, 2002; Constantino et al., 2017), show functional inter-dependency (Moriguchi, 2014; Fong and Iarocci, 2020), and for which individual differences tend to be relatively stable through development (in the absence of significant intervention; Frogner et al., 2022), these analyses were expected to elucidate important neurobehavioral correlates of challenging behavior. We hypothesized that multiple social and executive functioning domains, as well as emotion regulation and risk avoidance, would independently predict overall and specific types of challenging behavior, with emotion regulation being the strongest predictor. However, given the lack of prior evidence regarding the exact nature of specific relationships, no further specificity of relationships was hypothesized. The second aim was to examine how challenging behavior domains influence quality of life and other functional impacts. It was expected that challenging behavior would show significant and strong relationships with quality of life and functional impact measures. Lastly, exploratory models examined whether challenging behavior may mediate relationships between drivers (executive and social functioning domains) and impacts (quality of life and other functional impacts). Given that challenging behaviors occur in ASD, across other conditions and normative development, and that proposed risk factors and downstream effects are transdiagnostic rather than diagnosis specific, the current investigation sought to address noted questions using a large sample spanning normative and atypical development, including, but not constrained to ASD.

## Materials and methods

### Participants

Informants were recruited using the Prolific Academic online data collection service.^1^ The final sample was comprised of 2,004 valid surveys. Detailed information regarding the sample is available in our prior psychometric evaluation of the challenging behavior scale (Frazier et al., 2022). The study was reviewed and approved by the local institutional review board and all participants provided informed consent.

### Diagnostic information

Any (current/prior) developmental or neuropsychiatric diagnoses were also collected for each child, including: ASD, ADHD, and several other neurodevelopmental conditions. As the aims of this study were not focused on specific diagnostic groups, and diagnostic information from this sample has been previously reported (Frazier et al., 2022), ASD and other neurodevelopmental disorders were considered as a group for sensitivity analyses. This was done to examine whether significant deviations in the full sample pattern are seen in individuals with developmental conditions.

### Measures

#### Open source – challenging behavior scale

The OS-CBS is an 18-item informant-report questionnaire measure that was developed to evaluate challenging behavior in children with neurodevelopmental disorders. The measure includes five subscales: property destruction, aggression, elopement, conduct problems, and self-injury. Items were rated using a 5-point Likert scale with the following choices: 1 = “*not at all a problem*,” 2 = “*mild problem*,” 3 = “*moderate problem*,” 4 = “*severe problem*,” and 5 = “*very severe problem*.” For the present study, analyses focused on item average scores from challenging behavior total scale and the five subscales representing specific types of problem behavior.

#### Stanford social dimensions scale

The SSDS (Phillips et al., 2019) is a 71-item informant-report questionnaire measure that assesses social functioning including items evaluating social communication, social motivation, social affiliation, social recognition, and unusual social approach. Items are rated on a 5-point Likert scale using choices: “*never*,” “*rarely*,” “*sometimes*,” “*often*,” and “*always*.” Scores are summed across items and higher sum scores on the SSDS total score and subscales indicate better functioning, with the exception of unusual social approach where higher sum scores indicate greater atypicality.

#### Executive functioning scale

The EFS is a 52-item informant-report questionnaire measure that was created to improve the ability to assesses distinct executive functioning processes relative to existing measures (Uljarevic et al., in preparation). The instrument includes a total score and the following three core executive functioning subscales: sequencing/working memory, response inhibition, and set shifting. In addition to core executive functioning subdomains, similar to other widely used measures of executive functioning such as the Behavior Rating of Executive Function (Gioia et al., 2000), the EFS also captures distinct, yet related subdomains of emotion regulation and risk avoidance. Scoring was based on a set of exploratory structural equation analyses (Supplementary material 2). The final solution used in the present analyses included a general EF factor and specific sequencing/working memory, response inhibition, set shifting, emotion regulation, and risk avoidance factors. This solution had excellent fit (CFI = 0.963, TLI = 0.950, RMSEA = 0.057, 95% CI = 0.056–0.058). EFS items are rated on a 5-point Likert scale: “*never*,” “*rarely*,” “*sometimes*,” “*often*,” and “*very often*.” Item averages were used to score the total score and each subscale. The EFS total score shows good convergent validity with the 24-item BRIEF (LeJeune et al., 2010) and good discriminant validity with measures of other cognitive functions and psychopathology. Higher scores on the executive functioning total and subscale scores indicate better functioning (see Supplementary material 2 for additional psychometric information).

#### Child and family quality of life – Second edition

The CFQL-2 is a 26-item parent questionnaire that evaluates seven different aspects of child and family quality of life: child, family, caregiver, financial, external support, partner relationship, and coping quality of life (Frazier et al., 2020). Items use a 5-point Likert scale ranging from (1 = “*Strongly Disagree/Decreased Substantially*” to 3 = “*Neutral/Same*” *to 5 =* “*Strongly Agree/Improved Substantially*”). For the present study, total and subscale item average scores were included in analyses.

#### Functional impact measures

Participants also responded to nine questions attempting to elicit possible impacts of challenging behavior on parental work, the child’s participation in school, social interaction with peers/friends, community participation, contact with medical professionals, and legal interactions (Supplementary material 3). Bivariate non-parametric correlations among these questions revealed several very high correlations. For this reason, and to reduce the complexity of structural analyses for each study aim, principal components analysis was conducted to summarize these variables. Results indicated two eigenvalues surpassing 1.0 and the 95% confidence interval of Horn’s parallel analysis (Glorfeld, 1995; O'Connor, 2000). After promax rotation, the first component had high loadings from the parent work, child school, social, and community questions. The second component had high loadings from the medical and legal questions. The results of PCA were used to create composite scores by first standardizing each variable and the averaging variables with high loadings for each respective composite. These standardized average scores were then used in subsequent structural models to assess parent/child impacts and medical/legal impacts.

### Procedure

Prospective participants reviewed an electronic consent form. This form described that the purpose of this study was to evaluate several important domains of functioning relevant to individuals with neurodevelopmental disorders. They were told that they would answer questions about their identified child’s behavior and functioning. Participants who decided to continue indicated consent electronically and began the survey. All surveys were complete in Qualtrics and participants were paid US$10 for survey completion based on the expected completion time (35 min). All data were collected anonymously.

### Statistical analyses

The primary focus of all analyses was on relationships across measures of clinically-relevant domains and not measurement within a domain. For this reason, and to focus on how the domains are measured in practice, all analyses used unit-weighted observed indicators based on prior factor analyses. The unit-weighted scores were used to assess each of the major domains: social and executive functioning, challenging behavior, child and family quality of life, and functional impacts and the respective sub-domains.

To evaluate the first aim, concerning executive and social functioning influences on challenging behavior, two separate structural (path) models were computed with each SSDS and EF subscale as predictors and challenging behavior total and subscale scores as dependent variables in separate analyses. Specifically, the primary analysis focused on the executive and social functioning measures as predictors of challenging behavior total scores and the secondary analysis examined challenging behavior subscales.

To evaluate the second aim, concerning the influence of challenging behavior on quality of life and functional impacts, two structural models were estimated with challenging subscale scores as predictors and CFQL-2 total and subscale scores and functional impact scores as dependent variables in separate analyses. Specifically, the primary analysis focused on the challenging behavior subscales as predictors of CFQL-2 total and functional impact scores and the secondary analysis examined CFQL-2 subscales. Note: Aims 1 and 2 could be combined into a large structural model but these were kept separate for simplicity of presentation.

To evaluate aim 3, a mediational model was computed with EF and SSDS total scores as upstream predictor variables with direct effects to challenging behavior total score and to CFQL-2 total scores and functional impact scores. Challenging behavior total score also was estimated to have a direct effect on CFQL-2 total scores and functional impact scores. Indirect effects of EF and SSDS total scores on CFQL-2 total scores and functional impact scores through challenging behavior total scores were also estimated. The proportion of the total effect due to mediation was determined by dividing the standardized indirect effect by the standardized total effect (Ditlevsen et al., 2005).

For all models, maximum likelihood estimation was used with linear link functions. The predictive strength for dependent variables was evaluated using *R*^2^. Standardized estimates and the associated standard errors are presented to permit evaluation of the relative predictive strength for each variable. Effect size conventions were: small (*β* = 0.14), medium (*β* = 0.39), and large (*β* = 0.59) for standardized path coefficients and very weak (*R*^2^ < 0.02), weak (0.02 ≤ *R*^2^ < 0.13), moderate (0.13 ≤ *R*^2^ < 0.26), and substantial (*R*^2^ > =0.26) for overall prediction of dependent variables (Cohen, 1987). All analyses were conducted on cross-sectional data and, therefore, prediction is used only in the statistical sense. All models were just identified; however, model fit was also estimated after removing all non-significant paths to determine whether these paths are needed.

### Sensitivity analyses

To evaluate whether the pattern of results might change as a function of sample composition, a series of multi-group structural analyses were estimated based on the models used to evaluate each aim. Separate multi-group analyses were estimated to compare estimates between those with and without developmental disability diagnoses (diagnostic groups), male and female children (sex), and ages < 8 and ≥8 (age). In each case, an unconstrained model was fit to examine standardized parameter differences between groups and a constrained model where all structural parameters were held constant across groups (6 total models for each aim – 1 constrained and 1 unconstrained model were computed, separately for diagnostic groups, age, and sex).

The fit of the constrained model was evaluated using the Comparative fit index (CFI), Tucker–Lewis index (TLI), and root mean square error of approximation (RMSEA; Hu and Bentler, 1999; Marsh et al., 2004). Chi-square and its significant were also evaluated but this tends to be overly sensitive to deviations in fit in large sample sizes. For this reason, constrained models were only considered to show misfit if *p* < 0.001 and CFI or TLI were <0.90 or RMSEA was >0.08. To evaluate the magnitude of any statistically significant differences between subgroups, the average absolute difference in standardized coefficients was computed for each model. Bivariate correlations between the group coefficients were also calculated to explore whether the pattern of standardized coefficients was consistent across groups.

### Missing data handling

The only missing data occurred for the partner relationship scale of the CFQL-2. Nearly 15% of informants reported no significant partner relationship. The average correlation between missingness on this variable and the other variables included in this study was very small (*r* = −0.03) and there were no correlations above |*r*| = 0.16. Based on this pattern, the tolerance of structural models using maximum likelihood estimation for data approximating missing at random, and the possibility that excluding cases with missing data could bias results, all cases were included in analyses.

### Statistical power

Given the large sample size (*N* = 2,004) with complete data, it was anticipated that most statistical tests, including tests for standardized path coefficients would be over-powered, even at small effect sizes. To further estimate statistical power for aims 1 and 2, a simulation study (*K* = 1,000 samples, *N* = 2,004, *α* = 0.05, two-tailed) was conducted with 10 predictors (aim 1) or five predictors (aim 2) and five dependent variables (aim 1) or seven dependent variables (aim 2). Simulations were conservatively estimated with large residual correlations among predictors and dependent variables (*r* = 0.50). Results indicated very good power (>0.86) for detecting small standardized path coefficients (*β*s = 0.10, equivalent to *f*^2^ = 0.02) between predictor and dependent variables in both aim 1 and aim 2 scenarios.

To examine the power to simultaneously detect significance in the direct and indirect paths in the exploratory mediation model, a simulation study (*K* = 1,000 samples, *N* = 2,004, *α* = 0.05, two-tailed) was conducted where the direct path parameters (equivalent to *β* in a regression approach to path analysis) were estimated using a small effect size (standardized path coefficients *β*s = 0.10, equivalent to *f*^2^ = 0.02; creating indirect effects representing mediation = 0.01). Results indicated excellent power (>0.96) to simultaneously detect small direct and indirect effects.

Data preparation and distributional assumptions were checked used SPSS v28 (IBM Corp, 2021). All power simulation studies and structural models were computed in MPlus version 8.7 (Muthén and Muthén, 1998-2017). The study was not pre-registered. Data are available on request from the first author.

## Results

### Participant characteristics

The sample has been previously described (Frazier et al., 2022) and sample characteristics are also included in Supplementary material 4. Briefly, the sample included 2,004 children (ages 2–17; Supplementary material 4); 169 children with parent-reported diagnoses of ASD (Age *M* = 10.5, SD = 4.8, 76% male; 18% non-white race/ethnicity), 541 children with other developmental disability (DD) diagnoses (Age *M* = 11.4, SD = 4.6, 55% male, 17% non-white race/ethnicity), and 1,294 neurotypical (NT) children (Age *M* = 8.5, SD = 4.7, 48% male, 18% non-white race/ethnicity). Sex distributions followed the expected pattern with a ~3:1 male:female ratio in ASD diagnosed sub-sample and an enrichment of males having a reported ADHD diagnosis. Consistent with the existing literature (Simonoff et al., 2008), rates of co-occurring diagnoses were substantial in the developmental disability groups. Social functioning was lower in the ASD group, executive functioning was lower in all of the developmental disability groups and the ASD and ADHD groups showed highest levels of challenging behavior with other DD group being slightly elevated above the neurotypical group. Descriptive statistics for key variables included in subsequent structural models are provided in Supplementary material 5 and a correlation matrix among these variables is included in Supplementary material 6. All analyses are based on cross-sectional data.

### Aim 1: Executive functioning, emotion regulation, risk avoidance and social functioning on challenging behavior

**Figure 1:**
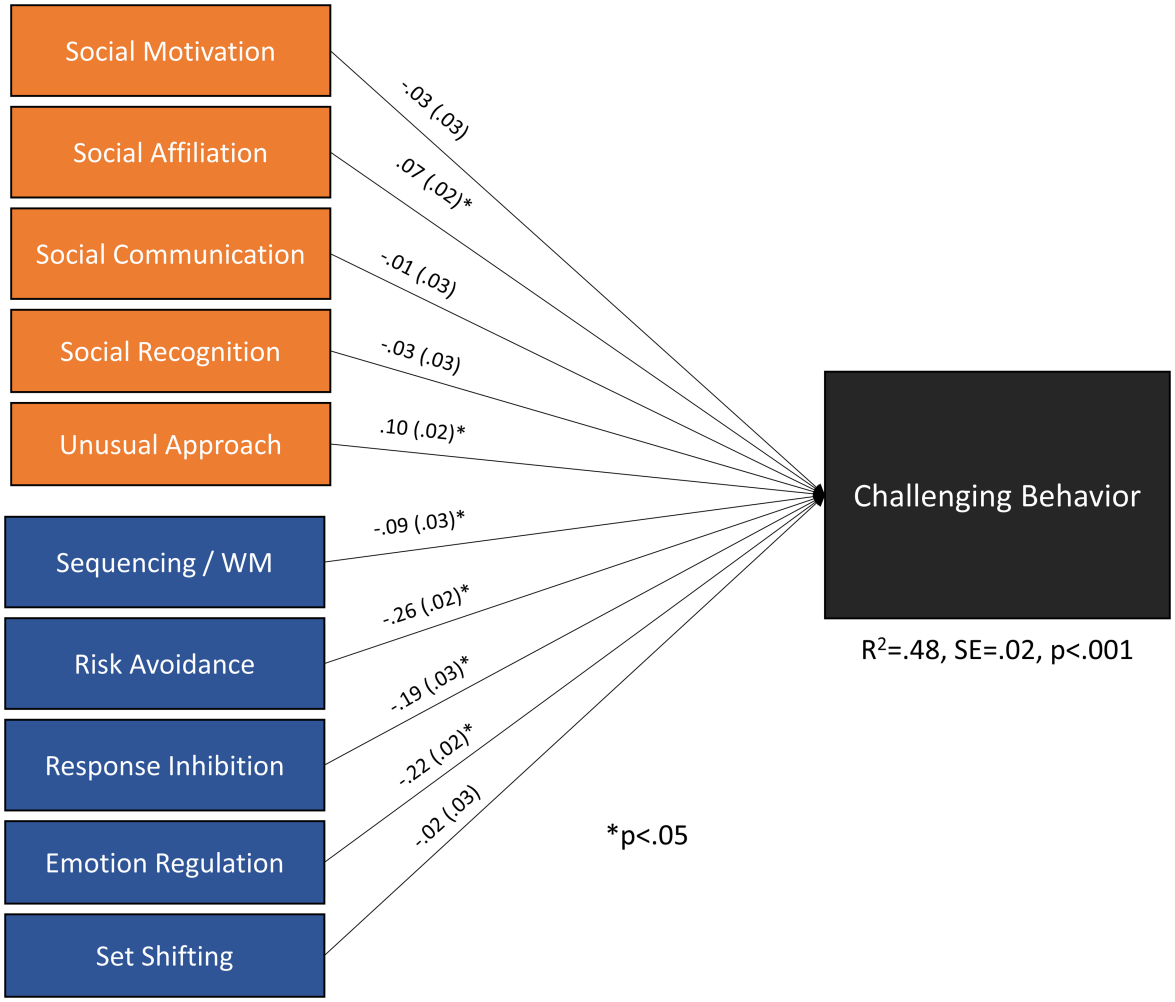
Executive and social functioning measures as predictors of challenging behavior. The model is just identified. Correlations among executive functioning subscales ranged from 0.40 to 0.76. Correlations among social functioning (SSDS) subscales ranged from |0.29| to |0.74|. The correlations between executive and social functioning subscales ranged from |0.07| to |0.63|. The pattern of residual correlations was consistent with the observed bivariate correlations among these subscales (see Supplementary material 6).

Higher levels of **s**equencing/working memory, risk avoidance, response inhibition, and emotional regulation were significantly independently associated with lower levels of total challenging behavior (Figure 1), with risk avoidance and emotion regulation showing the strongest relationships followed by response inhibition. Additionally, higher levels of social affiliation and unusual social approach were associated with higher levels of total challenging behavior. These individual relationships were small to medium-sized (|*β*| = 0.07–0.26) in magnitude. Overall prediction of challenging behavior was substantial, accounting for nearly half the variance (*R*^2^ = 0.48); excluding social functioning indicators from the model only reduced predictive strength very slightly (*R*^2^ = 0.47), indicating executive function processes play a substantial part in challenging behavior. Removing non-significant paths did not substantially reduce model fit [*χ*^2^(4) = 5.9, *p* = 0.2072, RMSEA = 0.015, CFI = 0.999, TLI = 0.996].

A similar pattern was observed when evaluating associations with the five challenging behavior subscales with a few exceptions (Table 1). Sequencing/working memory had weaker associations with property destruction, aggression, and self-injury; response inhibition was not significantly associated with elopement or self-injury; set shifting was significantly associated with self-injury (but not other subscales); and social affiliation was not significantly related to elopement or self-injury (but was for the other subscales). Predictive strength was variable across subscales, ranging from medium-sized for self-injury (*R*^2^ = 0.17) to very large for conduct problems (*R*^2^ = 0.51).

**Table 1:** Standardized path coefficients from executive and social functioning measures to challenging behavior subscales.

	Property destruction	Aggression	Elopement	Conduct problems	Self-injury
	*β* (*p*)	*β* (*p*)	*β* (*p*)	*β* (*p*)	*β* (*p*)
*Social functioning*					
Social motivation	−0.01 (0.956)	0.01 (0.908)	−0.04 (0.174)	−0.02 (0.410)	−0.09 (0.013)
Social affiliation	0.10 (<0.001)	0.06 (0.030)	−0.02 (0.487)	0.10 (<0.001)	0.01 (0.942)
Social communication	−0.03 (0.320)	−0.06 (0.082)	0.04 (0.242)	0.03 (0.254)	−0.05 (0.167)
Social recognition	−0.01 (0.633)	−0.02 (0.599)	−0.16 (<0.001)	−0.01 (0.731)	0.05 (0.111)
Unusual social approach	0.09 (<0.001)	0.08 (0.002)	0.10 (<0.001)	0.08 (<0.001)	0.06 (0.014)
*Executive functioning*					
Sequencing/Working memory	−0.07 (0.015)	0.01 (0.934)	−0.17 (<0.001)	−0.11 (<0.001)	−0.02 (0.661)
Risk avoidance	−0.22 (<0.001)	−0.18 (<0.001)	−0.32 (<0.001)	−0.20 (<0.001)	−0.12 (<0.001)
Response inhibition	−0.22 (<0.001)	−0.16 (<0.001)	−0.01 (0.692)	−0.27 (<0.001)	0.05 (0.106)
Emotion regulation	−0.23 (<0.001)	−0.22 (<0.001)	0.07 (0.017)	−0.27 (<0.001)	−0.17 (<0.001)
Set shifting	0.04 (0.281)	−0.01 (0.916)	−0.01 (0.985)	0.01 (0.998)	−0.14 (0.001)
	*R*^2^ = 0.39	*R*^2^ = 0.27	*R*^2^ = 0.29	*R*^2^ = 0.51	*R*^2^ = 0.17

All *R*^2^, SE = 0.02 and *p* < 0.001. Residual correlations for this model were: social motivation with social affiliation *r* = 0.64, social communication *r* = 0.74, social recognition *r* = 0.50, unusual social approach *r* = −0.48; social affiliation with social communication *r* = 0.70, social recognition *r* = 0.51, unusual social approach *r* = −0.29; social communication with social recognition *r* = 0.68 and unusual approach *r* = −0.42; and social recognition with unusual social approach *r* = −0.46; sequencing/working memory with risk avoidance *r* = 0.40, response inhibition *r* = 0.69, emotion regulation *r* = 0.52, set shifting *r* = 0.76, social motivation *r* = 0.38, social affiliation *r* = 0.37, social communication *r* = 0.49, social recognition *r* = 0.63, and unusual approach *r* = −0.41; risk taking with response inhibition *r* = 0.52, emotion regulation *r* = 0.44, set shifting *r* = 0.44, social motivation *r* = 0.11, social affiliation *r* = 0.07, social communication *r* = 0.17, social recognition *r* = 0.33, and unusual social approach *r* = −0.34; response inhibition with emotion regulation *r* = 0.60, set shifting *r* = 0.71, social motivation *r* = 0.31, social affiliation *r* = 0.30, social communication *r* = 0.41, social recognition *r* = 0.58, and unusual social approach = −0.40; emotion regulation with set shifting *r* = 0.71, social motivation *r* = 0.42, social affiliation *r* = 0.24, social communication *r* = 0.40, social recognition *r* = 0.40, unusual social approach *r* = −0.44; set shifting with social motivation *r* = 0.50, social affiliation *r* = 0.38, social communication *r* = 0.52, social recognition *r* = 0.58, and unusual social approach *r* = −0.51.

### Aim 2: Challenging behavior on quality of life and functional impacts

**Figure 2:**
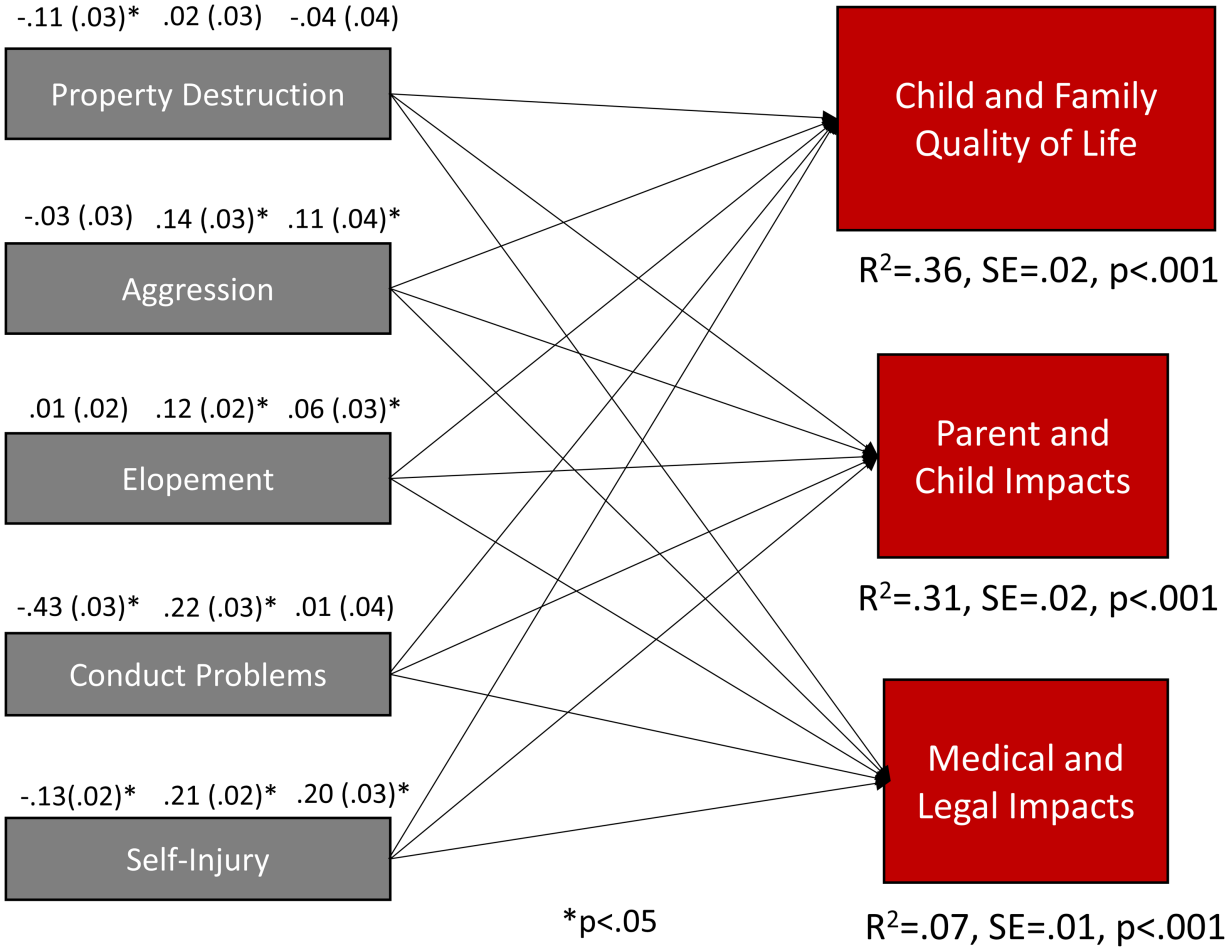
Challenging behavior subscales as predictors of child and family quality of life, work/school, and medical/legal functional impacts. The model is just identified. For each challenging behavior subscale, standardized parameters are listed in order starting with CFQL-2, Parent and Child Impacts, and Medical and Legal Impacts. Correlations among challenging behavior subscales ranged from 0.39 to 0.76. Correlations among the CFQL-2 and impact subscales were (CFQL-2 with parent/child impacts *r* = −0.41; CFQL-2 with medical/legal impacts *r* = −0.09; parent/child impacts with medical/legal impacts *r* = 0.22). The pattern of residual correlations was consistent with the observed bivariate correlations among these subscales (see Supplementary material 6). Gray boxes are challenging behavior scales serving as predictors, while red boxes are quality of life and impact outcome variables.

Higher levels of property destruction, conduct problems, and self-injury (but not aggression or elopement) were associated with lower overall levels of child and family quality of life (Figure 2 – right side). Aggression, elopement, and self-injury were significantly associated with parent/child and medical/legal impacts; conduct problems was only significantly associated with parent/child impacts (not medical/legal impacts); and property destruction was not associated with either type of functional impact (Figure 2 – left side). These individual relationships were generally small to medium (|*β*| = 0.06–0.43) in magnitude. Overall prediction of child and family quality of life (*R*^2^ = 0.36) and parent/child impacts (*R*^2^ = 0.31) was substantial, while prediction of medical/legal impacts was weak (*R*^2^ = 0.07). Removing non-significant paths did not reduce model fit [*χ*^2^(5) = 3.6, *p* = 0.613, RMSEA = 0.000, CFI = 1.0, TLI = 1.0].

When examining child and family quality of life subscales, several unique patterns emerged (Table 2). Property destruction was only significantly associated with child, family, caregiver, and coping quality of life subscales (not financial, external support, or partner relationship). Self-injury was significantly associated with all subscales except caregiver and partner relationship. In spite of not being significantly independently associated with total challenging behavior, aggression was significantly related financial and coping subscales and elopement was related to child, external support and coping subscales; indicating highly specific relationships between challenging behavior types and specific aspects of child and family quality of life.

**Table 2:** Standardized path coefficients from challenging behavior subscales to quality of life subscales.

	Child QoL	Family QoL	Caregiver QoL	Financial QoL	External support QoL	Partner relationship QoL	Coping QoL
	*β* (*p*)	*β* (*p*)	*β* (*p*)	*β* (*p*)	*β* (*p*)	*β* (*p*)	*β* (*p*)
Property destruction	−0.10 (0.004)	−0.15 (<0.001)	−0.14 (<0.001)	−0.05 (0.198)	−0.04 (0.270)	0.01 (0.942)	−0.09 (0.008)
Aggression	0.03 (0.295)	−0.04 (0.174)	−0.05 (0.084)	−0.07 (0.045)	0.03 (0.369)	−0.02 (0.620)	−0.06 (0.044)
Elopement	0.08 (0.001)	−0.01 (0.888)	0.04 (0.052)	−0.05 (0.038)	−0.10 (<0.001)	0.03 (0.348)	0.08 (<0.001)
Conduct problems	−0.39 (<0.001)	−0.02 (<0.001)	−0.43 (<0.001)	−0.17 (<0.001)	−0.20 (<0.001)	−0.23 (<0.001)	−0.38 (<0.001)
Self-injury	−0.13 (<0.001)	−0.08 (<0.001)	−0.03 (0.222)	−0.15 (<0.001)	−0.13 (<0.001)	−0.05 (0.082)	−0.11 (<0.001)
	*R*^2^ = 0.23	*R*^2^ = 0.36	*R*^2^ = 0.34	*R*^2^ = 0.16	*R*^2^ = 0.13	*R*^2^ = 0.06	*R*^2^ = 0.28

All *R*^2^, SE ≤ 0.02 and *p* < 0.001. Residual correlations for this model were: property destruction with aggression *r* = 0.28, elopement *r* = 0.19, conduct problems *r* = 0.40, self-injury *r* = 0.12; aggression with elopement *r* = 0.13, conduct problems *r* = 0.27, self-injury *r* = 0.08; elopement with conduct problems *r* = 0.21 and self-injury *r* = 0.08; and conduct problems with self-injury *r* = 0.12; child and family quality of life with parent and child impacts *r* = −0.11 and medical and legal impacts *r* = −0.03; and parent and child impacts with medical and legal impacts *r* = 0.10.

### Aim 3: Mediational model

**Figure 3:**
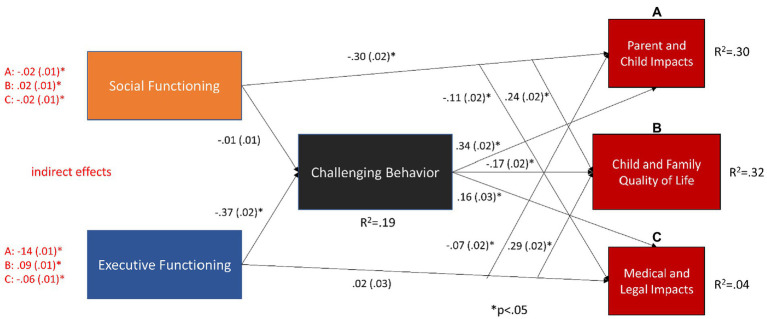
Mediational model estimating direct effects of executive and social functioning on quality of life and functional impacts as well as indirect effects *via* challenging behavior. The model is just identified. Branching arrows represent direct effects from social and executive functioning to child and family quality of life and functional impact measures. Red font designates indirect effects from social or executive functioning to parent and child impacts **(A)**, child and family quality of life **(B)**, or medical and legal impacts **(C)**. Residual correlations were as follows: social functioning with executive functioning *r* = 0.38; CFQL-2 with Parent and Child Impacts *r* = −0.26; CFQL-2 with Medical and Legal Impacts *r* = −0.08; Parent and Child Impacts with Medical and Legal Impacts *r* = 0.26.

There were significant direct effects from social and executive functioning to quality of life and functional impact measures prior to estimating the mediational model, with the exception of the direct effect of executive functioning on medical and legal impacts (*p* = 0.111). Executive functioning showed a significant direct effect on child and family quality of life but not functional impact measures (Figure 3). Interestingly, however, all indirect effects from executive functioning to quality of life and impact measures *via* challenging behavior were significant, accounting for substantial proportions of the total effects (% of total effect: quality of life – 23%; parent/child impact – 67%; and medical/legal impact – 97%). All of the indirect effects represent partial mediation with the exception of the indirect effect of executive functioning on medical and legal impacts *via* challenging behavior which represents full mediation. Reduction of the magnitude of significant direct effects when including indirect effects tended to be modest (<25% reduction in the magnitude of the direct effect).

Social functioning had significant direct effects to child and family quality of life and functional impact measures. Small but significant indirect effects were also observed from social functioning to all three dependent variables *via* challenging behavior.

Removing the non-significant direct path from executive functioning to medical and legal impacts did not reduce model fit [*χ*^2^(1) = 0.6, *p* = 0.439, RMSEA = 0.000, CFI = 1.0, TLI = 1.0].

### Sensitivity analyses

Multi-group analyses for aim 1 found no significant misfit for the age or sex subgroups (Supplementary material 7). Misfit was significant for the diagnostic groups model with the two major differences being a weaker effect of sequencing/working memory on challenging behavior in the DD group and a stronger influence of unusual social approach on challenging behavior in the DD group. Even with these differences, the average absolute standardized coefficient differences were modest (|0.07|).

Multi-group models for aim 2 all showed significant misfit (Supplementary material 8). For diagnostic groups, the major differences were a non-significant influence of aggression on quality of life in the DD group, a significant negative effect of self-injury on quality of life. For sex, the largest differences were that the effect of aggression on quality of life was no longer significant in males, the effect of elopement and parent/child impact was significant in males (but not females), and the effect of self-injury on parent/child impact was strong in females but much weaker in males. Even with these differences, the average absolute standardized coefficient differences were modest (|0.08|).

Multi-group models for aim 3 found significant misfit for diagnostic groups and age but not sex (Supplementary material 9). For diagnostic groups, the differences in coefficients all tended to be small (average absolute difference = |0.04|) except for the direct effect from the executive functioning scale to parent/child impact which was weaker and non-significant in the DD group. For age, the direct effects from executive and functioning to child and family quality of life were stronger in the 8 and older age group. Similarly, the direct and indirect effects from executive functioning to parent/child impacts were stronger in the 8 and older age group. However, the average absolute difference in standardized coefficients was small (|0.04|).

For all multi-group models, the patterns of standardized coefficient loadings were highly correlated, consistent with small and inconsistent absolute differences (all *r* ≥ 0.76).

No cases of regression suppression effects were detected for any of the primary or sensitivity analyses.

## Discussion

To our knowledge, this is the first large scale structural analysis of possible neurobehavioral drivers (executive and social functioning, including emotion regulation and risk avoidance) and impacts (quality of life and work/school/medical/legal impacts) of challenging behavior in children and adolescents. Although challenging behaviors are particularly common in ASD, they also frequently occur across other neurodevelopmental disorders and can occur in children and adolescents without any specific diagnosis. Regardless of the specific diagnosis, challenging behaviors are highly impairing and present significant management challenges. Importantly, previous literature has suggested that both risk factors and downstream effects of challenging behaviors are not specific to any particular disorder. Thus, in this investigation, we focused on a transdiagnostic sample comprising children and adolescents with and without neurodevelopmental disabilities. Four main findings emerged from this investigation:

1. Executive functioning skills (particularly response inhibition), emotion regulation and risk avoidance were strongly associated with lower levels of challenging behavior, accounting for nearly half the variance in challenging behavior total scores.
2. Challenging behaviors were associated with significant reductions in child and family quality of life, negative impacts on parent/child functioning, and greater interactions with the medical/legal systems, accounting for over 30% of the variance in quality of life and parent/child functioning.
3. Executive functioning, emotion regulation, risk avoidance and social functioning show direct effects on quality of life and parent/child/medical/legal impacts as well as indirect effects *via* challenging behavior.
4. Each of the above described broad effects aggregate interesting patterns of more specific effects between facets of executive and social functioning, challenging behavior, and quality of life that have implications for future research and clinical practice and are discussed in turn.

The individual effects of risk avoidance and emotion regulation on challenging behavior were substantial and suggest that executive functions, including the related domains of emotion regulation and risk avoidance, play a key role the manifestation and/or maintenance of challenging behavior. Interestingly, sequencing/working memory seems to only play a role in elopement and conduct problems, while set shifting was only associated with self-injury and response inhibition was not significantly related to elopement or self-injury behaviors. Research is needed to better understand the developmental interplay of specific executive functions on the emergence and ongoing occurrence of challenging behavior. The possibility that early problems in the development of specific executive functions increases the likelihood of future challenging behavior, if identified, could present both the opportunity to identify at-risk children early but also the potential to develop tailored prophylactic interventions to avoid to onset of these behaviors. These identified patterns for individual executive functions also suggests that, rather than only collecting aggregate measures, future longitudinal studies would benefit from focusing on specific executive function processes while quantifying different types of challenging behaviors (Northrup et al., 2022).

Clinically, the present findings indicate that intervention strategies for each of the challenging behavior types would benefit from considering both the functional contributions (ex. attention, escaping demands, gaining a desired object, etc.), determined *via* behavioral assessment, and the set of executive functions that are likely influencing the development and maintenance of the behavior. For example, if associations between self-injury and risk avoidance, emotion regulation, and set shifting are replicated in future work, interventions might be more effective if they not only focus on standard behavior replacement strategies but also, where possible, to supplement these with teaching strategies with differential reinforcement for managing emotions, being flexible with transitions, and identifying risky versus safe behaviors. Thus, the present data suggest that clinicians evaluating challenging behavior would benefit from including a measure of executive function to develop a more comprehensive and tailored intervention approach.

The present results are consistent with the growing literature identifying a prominent role for emotion dysregulation in the occurrence of challenging behavior (Berkovits et al., 2017). Prior work has suggested that this domain may be essential for minimizing the occurrence of challenging behavior. The present findings strongly support this notion but also provide the qualification that emotion dysregulation may be less important for elopement behaviors. Instead, these may be largely driven by problems in understanding and using risk information and to a lesser extent the ability to order information and hold it in mind. The latter association is interesting and suggests that at least some instance of elopement may be, in part, driven by difficulty with sustaining mental context and having attention easily drawn to a desired object or activity. While not anticipated *a priori*, this interpretation does comport with observations of some young children with autism spectrum disorder who elope when seeking something they like or an activity they want to engage in, including leading to tragedies such as drowning or getting lost during winter months. While generalizing executive functioning improvements during training to the real-world has proved challenging (Faja et al., 2022), developing tailored intervention strategies for challenging behavior that include accommodating executive functioning deficits and using the executive functioning profile in the context of treatment planning should be explored to reduce the likelihood of these impairing, and often dangerous, behaviors.

In contrast to the strong associations for executive functioning, the effects of social functions were either non-significant or much weaker. Notably, the effects of affiliation and unusual social approach while statistically significant, accounted for very variance in challenging behavior scores. Further, the direction of the relationship for social affiliation is counter-intuitive. Together, these effects suggest that opportunities for interaction (affiliation) and lack of appropriate engagement (unusual approach) play small roles in the occurrence of challenging behavior problems. Specifically, greater opportunities for interaction appears to be a double-edged sword, allowing children – particularly those with autism and other developmental disabilities – to learn social skills and develop close relationships, but also increasing the likelihood of negative behaviors and interactions. This highlights to need, particularly in young children at risk for challenging behavior, to ensure that intervention and active teaching are used before and during social opportunities to ensure that positive interactions and relationships result.

Several specific associations between types of challenging behavior and facets of child and family quality of life emerged in this study. Interestingly, interpreting these effects using Bronfebrenner’s ecological systems theory (Bronfenbrenner, 1989), property destruction tends to most impact the quality of life of the individual and microsystem (child, family, caregiver, and coping), while not impacting functionally; no associations were observed with child/family or medical/legal impacts. In contrast, self-injury has effects on the quality of life of the individual and microsystem but also on the mesosystem and exosystem (financial and external support quality of life and medical/legal impacts). Most prominently, conduct problems had the strongest and most consistent effects on the whole ecological system. This suggests that oppositional and conduct issues may be one of the most important types of challenging behaviors to identify and intervene on early in life to reduce its future occurrence and impact. The complexity of relationships between challenging behavior types and impacts, including quality of life and functional impacts, further reinforces the need for longitudinal research that can account for temporal aspects of prediction and identify likely causative relationships. Clinically, this complexity also reinforces the need to evaluate specific types of challenging behavior and include a multi-faceted intervention plan that considers the child and family but also the larger ecosystems in which the child participates.

Mediational model results further elucidate the cross-sectional complexity of relationships between executive and social functioning, challenging behavior, quality of life, and functional impacts. Most notably, while social functioning provides minor incremental contributions to challenging behavior, its associations with quality of life and functional impacts were predominantly direct (not through challenging behavior). In contrast, executive functioning showed both direct and indirect effects (*via* challenging behavior) on quality of life and functional impacts. Clinically, if these results are extended in longitudinal data, this may suggest that attempts to improve quality of life and decrease functional impacts in children and families should consider executive and social functioning as well as challenging behavior. However, specific attempts to reduce challenging behavior may best focus effort on how executive functions and emotion regulation could incrementally influence the manifestation of challenging behavior with less attention to social functioning.

## Limitations and future directions

As a cross-sectional study relying on questionnaire measures of neurobehavior, it is crucial that the present results be replicated in longitudinal samples using more objective measures of executive and social functioning and with extension to variations in sample composition. The need for longitudinal evaluation is particularly relevant for understanding mediational relationships between executive and social functioning, challenging behavior, quality of life, and functional impacts. It is very possible that these relationships are bidirectional or, in some case, in the opposite direction of how they have been specified in these cross-sectional analyses (Donati et al., 2021). Future investigations will be most clarifying if they include sufficient temporal and construct/domain granularity to look for cross-lagged associations with greater potential to represent causative relationships. Recent data suggest that more detail may be required even within domains. For example, one study found that different patterns of emotion dysregulation may manifest in children with autism spectrum disorder relative to peers (Northrup et al., 2021). In addition to greater granularity and including other measures of executive functions and challenging behaviors, collection of objective measures will be important for ensuring that identified relationships are not simply a function of subjective interpretations or rater biases.

As an informant-report study, it was also not possible to tease out whether differential relationships might be observed for the frequency and intensity of challenging behavior, as the challenging behavior scale uses only a problem rating framework. Thus, if feasible, future work should capture direct behavioral observations, including frequency counts and intensity descriptions of each challenging behavior type. This will require extensive monitoring but will be essential for further understanding how executive functions influence the topography of challenging behavior. Because some challenging behaviors are infrequent and direct behavioral observation is often expensive, it may be possible to use ecological momentary assessment methods to get more detailed information without prohibitive cost.

An additional limitation of this study is the focus on executive and social functions, including emotion regulation, as drivers of challenging behavior and the relatively limited assessment of functional impacts. While the present study chose executive and social functions based on prior literature (Maddox et al., 2018; Hukkelberg et al., 2019; Marleau et al., 2021) and clinical experience with children who exhibit high levels of challenging behavior (Frazier et al., 2010), future research should consider other potential influences such as general cognitive ability, speech/language/communication (Neuhaus et al., 2022), sleep disruption (Madrid-Valero et al., 2019), repetitive behavior and sensory processing (Griffin et al., 2022), adverse events (Baiden et al., 2017), and demographic or social/cultural factors (Neuhaus et al., 2022). In prior analyses with the present data (Vasudevan et al., in preparation), parent-estimated cognitive ability (often based on clinical test results) and speech/language level did not independently influence challenging behavior when executive functioning was included in the model, and sociodemographic factors had only limited incremental predictive validity for challenging behavior, it is possible that other factors play an important role.

Given the online nature of the study, it was not possible to verify parent-reported diagnoses using the gold standard diagnostic assessment methods. Further, the number of children and adolescents with ASD was relatively low. It will thus be of crucial importance for future studies to replicate and extend findings reported here by utilizing clinically referred sample of people with ASD and non-ASD conditions and to explore potential continuities and discontinuities in the mechanisms behind challenging behaviors and in mediators and moderators of their downstream effects on individuals, family system and broader social context.

## Conclusion

Notwithstanding noted limitations, the present findings provide important insights into the correlates of challenging behaviors by conducting the first comprehensive characterization of specific aspects of executive functioning domain as well as emotion regulation that serve as key drivers of challenging behavior. This study has also highlighted the major impact of challenging behavior on quality of life and functional impacts on the child, family, and larger social system. If replicated in future studies, these relationships can be used to better tailor intervention strategies for challenging behavior to maximize downstream effects on quality of life, particularly in children and families with neurodevelopmental conditions.

## Data availability statement

The raw data supporting the conclusions of this article will be made available by the authors, without undue reservation.

## Ethics statement

The studies involving human participants were reviewed and approved by John Carroll University – Institutional Review Board. The patients/participants provided their written informed consent to participate in this study.

## Author contributions

TF, MU, and AK designed the study. TF and MU oversaw data collection. TF and MU had full access to the data and conducted the analyses. TF drafted the initial manuscript. All authors contributed to the article and approved the submitted version.

## Funding

This study was funded, in part, by Autism Speaks (#12776) and the PTEN Research Foundation (JCU-20-001).

## Conflict of interest

Beyond the scope of this research, TF has received funding or research support from, acted as a consultant to, received travel support from, and/or received a speaker’s honorarium from the PTEN Research Foundation, SYNGAP Research Fund, Malan Syndrome Foundation, ADNP Kids Research Foundation, Quadrant Biosciences, Autism Speaks, Impel NeuroPharma, F. Hoffmann-La Roche AG Pharmaceuticals, the Cole Family Research Fund, Simons Foundation, Ingalls Foundation, Forest Laboratories, Ecoeos, IntegraGen, Kugona LLC, Shire Development, Bristol-Myers Squibb, Roche Pharma, MaraBio, National Institutes of Health, and the Brain and Behavior Research Foundation and has an investor stake in Autism EYES LLC and iSCAN-R. EC received research support funding from Autism Speaks.

The remaining authors declare that the research was conducted in the absence of any commercial or financial relationships that could be construed as a potential conflict of interest.

## Publisher’s note

All claims expressed in this article are solely those of the authors and do not necessarily represent those of their affiliated organizations, or those of the publisher, the editors and the reviewers. Any product that may be evaluated in this article, or claim that may be made by its manufacturer, is not guaranteed or endorsed by the publisher.

## Footnotes

^1^ https://prolific.co/
