# Supplementary material for: Associations between executive functioning, challenging behavior, and quality of life in children and adolescents with and without neurodevelopmental conditions
Source: Front Psychol. 2022 Oct 20;13:1022700. doi: 10.3389/fpsyg.2022.1022700 (PMC9632446; doi:10.3389/fpsyg.2022.1022700)
Supplement: Supplementary file 1 [file Table_1.DOCX]

**Online Supplement 1.** Study schema.

**
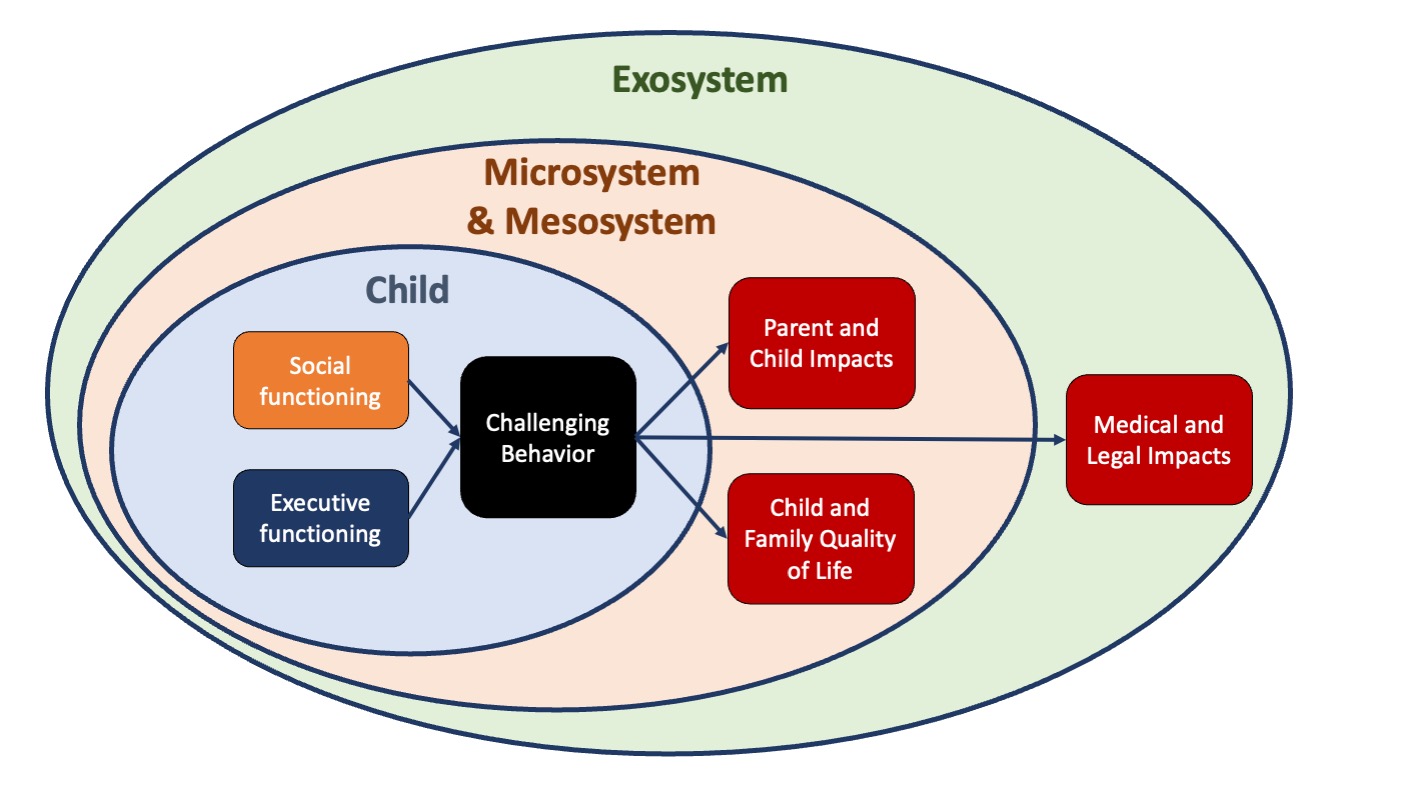
**

**Supplement 2.** Psychometric evaluation results for the executive functioning scale (EFS).

The EFS was developed as part of a larger effort to develop new informant-report measures for evaluation of children and adolescents with neurodevelopmental disorders and neurogenetic syndromes. The initial measure was developed with 36 items. Preliminary principal components analyses and exploratory factor models suggested 4 factors, however, the structure was not replicable across subsamples and several items demonstrated substantial cross-loadings. For this reason, the measure was revised and additional items were written, resulting in the final 52-item version used for the present study.

Initial psychometric analyses of this 52-item measure were conducted using exploratory structural equation modeling in training (n=2004) and testing subsamples (n=954). Analyses identified a solution with six specific factors and a general bifactor as showing excellent fit with interpretable loading patterns. Adding a seventh specific factor resulted in unstable solutions across subsamples and the last factor was not interpretable. Fit of this model in the total sample was excellent (CFI=.963, TLI=.950, RMSEA=.057, 95% CI=.056-.058, SRMR=.021).

Convergent and discriminant validity analyses were also conducted with the EFS and several other measures, including the 24-item Behavior Rating Inventory of Executive Function (BRIEF) [convergent validity) and measures of adverse childhood experiences (ACES), the modified overt aggression scale (MOAS), parent-reported autism symptom severity, and the other measures included in the present study (challenging behavior scale [CBS], Stanford social dimensions scale [SSDS], child and family quality of life [CFQL-2] scale, and the parent/child and medical/legal impact measures).

Results indicated good convergent validity between the EFS and the BRIEF (r=.85). The EFS also showed good discriminant validity as evidenced by lower correlations with other measures (EFS with ACES r=-.20, MOAS r=-.52, autism symptom severity r=-.45, SSDS r=.60, CBS r=-.66, CFQL-2 r=.72, parent/child impact r=-.49, medical/legal impact r=-.14). All of these correlations were statistically significantly lower than the correlation with the BRIEF (p<.001).

**Online Supplement 3**. Functional impact questions.

Parent Work

*1. How many hours of work have you or a family member missed in the past month due to your child / adolescent's challenging behaviors?*

0 hours

1 to 2 hours

3 to 4 hours

5 to 10 hours

11 to 15 hours

16 to 20 hours

More than 20 hours

*2. Have you or another family member had to reduce the number of typical working hours due to your child / adolescent's challenging behaviors?*

Yes

No

*3. Have you or another family member had to stop working due to your child / adolescent's challenging behaviors?*

Yes

No

Child School

*4. Has your child / adolescent ever been retained in their grade, experienced suspension (in- or out-of-school), or otherwise had a change in their school experience due to challenging behaviors?*

Yes

No

*5. How often has the child / adolescent missed school in the last month due to challenging behaviors?*

No days missed

1-2 days missed

3-5 days missed

6-10 days missed

More than 10 days missed

Social

*6. How would you rate the impact of your child / adolescent's challenging behaviors on their ability to make and / or keep friends?*

Extreme impact

Significant impact

Slight impact

No impact

Community

*7. How would you rate the impact of your child / adolescent's challenging behaviors on participating in school or community activities?*

Extreme impact

Significant impact

Slight impact

No impact

Medical

*8. How frequently in the past year has your child / adolescent visited the ER or has been hospitalized as a result of challenging behaviors?*

None

1-2 times

3-5 times

6-10 times

More than 10 times

Legal

*9. How often in the past year has your child / adolescent had an interaction with law enforcement officers as a result of challenging behaviors?*

None

1-2 times

3-5 times

6-10 times

More than 10 times

**Online Supplement 4.** Sample demographic and clinical characteristics.

|  | **ASD** | **DD** | **NT** | **X^2^ / F (p)** |
| --- | --- | --- | --- | --- |
|  | n (%) | n (%) | n (%) |  |
| **N** | 169 | 541 | 1294 |  |
| **Informant Age** |  |  |  | 43.1 (<.001) |
| 18 to 24 | 4 (2%) | 4 (1%) | 43 (3%) |  |
| 25 to 34 | 43 (25%) | 124 (23%) | 416 (32%) |  |
| 35 to 44 | 70 (41%) | 229 (42%) | 531 (41%) |  |
| 45 to 54 | 42 (25%) | 137 (25%) | 230 (18%) |  |
| 55 to 64 | 9 (5%) | 42 (8%) | 59 (5%) |  |
| 65 to 74 | 1 (0%) | 5 (1%) | 13 (1%) |  |
| 75 or older | 0 (0%) | 0 (0%) | 2 (1%) |  |
| **Informant Sex** |  |  |  | 39.9 (<.001) |
| Male | 37 (22%) | 135 (25%) | 445 (34%) |  |
| Female | 129 (76%) | 406 (75%) | 847 (66%) |  |
| Did not report | 3 (2%) | 0 (0%) | 2 (1%) |  |
| **Informant Relationship Status** |  |  |  | 37.8 (<.001) |
| Married | 95 (56%) | 358 (66%) | 953 (74%) |  |
| Single, never married | 19 (11%) | 51 (9%) | 91 (7%) |  |
| Divorced, separated, widowed | 37 (22%) | 71 (13%) | 118 (9%) |  |
| Single, cohabitating | 18 (11%) | 61 (11%) | 128 (10%) |  |
| **Household Income (US $)** |  |  |  | 40.9 (<.001) |
| <$25,000 | 27 (16%) | 65 (12%) | 103 (8%) |  |
| $25,000-$34,999 | 19 (11%) | 58 (11%) | 116 (9%) |  |
| $35,000-$49,999 | 29 (17%) | 64 (12%) | 137 (11%) |  |
| $50,000-$74,999 | 34 (20%) | 122 (23%) | 278 (21%) |  |
| $75,000-$99,999 | 26 (15%) | 96 (18%) | 218 (17%) |  |
| $100,000-$149,999 | 18 (11%) | 88 (16%) | 272 (21%) |  |
| $150,000-$199,999 | 9 (5%) | 27 (5%) | 88 (7%) |  |
| $200,000+ | 5 (3%) | 18 (3%) | 68 (5%) |  |
| Did not report | 2 (1%) | 3 (1%) | 14 (1%) |  |
| **Child Age** (M, sd) | 10.5 (4.8) | 11.4 (4.6) | 8.5 (4.7) | 77.4 (<.001) |
| **Child Sex** |  |  |  | 54.1 (<.001) |
| Male | 128 (76%) | 299 (55%) | 617 (48%) |  |
| Female | 41 (24%) | 241 (44%) | 674 (52%) |  |
| Intersex | 0 (0%) | 1 (1%) | 0 (0%) |  |
| Did not report | 0 (0%) | 0 (0%) | 3 (<1%) |  |
| **Child Race / Ethnicity** |  |  |  |  |
| White / Caucasian | 138 (82%) | 450 (83%) | 1065 (82%) | 0.3 (.865) |
| Black / AA | 18 (11%) | 52 (10%) | 118 (9%) | 0.5 (.795) |
| Middle Eastern or North African | 1 (1%) | 4 (1%) | 2 (<1%) | 4.1 (.131) |
| East Asian | 3 (2%) | 8 (2%) | 45 (4%) | 6.3 (.042) |
| South Asian | 2 (1%) | 1 (<1%) | 21 (2%) | 6.7 (.036) |
| Native American / Alaskan Native | 4 (2%) | 14 (3%) | 18 (1%) | 3.4 (.179) |
| Native Hawaiian / Pacific Islander | 1 (1%) | 4 (1%) | 6 (1%) | 0.5 (.764) |
| Multi-racial | 18 (11%) | 50 (9%) | 104 (8%) | 1.7 (.425) |
| Hispanic | 22 (13%) | 74 (14%) | 139 (11%) | 6.9 (.141) |
| Unknown | 0 (0%) | 2 (<1%) | 3 (<1%) | 0.8 (.686) |
| Did not report | 1 (1%) | 1 (<1%) | 11 (1%) | 2.6 (.269) |
| **Autism Severity Level** (informant-rated) |  |  |  |  |
| Mild | 82 (48%) | - | - |  |
| Moderate | 74 (44%) | - | - |  |
| Severe | 13 (8%) | - | - |  |
| **Cognitive Level** (informant-estimated) |  |  |  | 347.5 (<.001) |
| Very high or above (120+) | 20 (12%) | 59 (11%) | 131 (10%) |  |
| High Average (110-119) | 39 (23%) | 188 (35%) | 579 (45%) |  |
| Average (90-109) | 51 (30%) | 216 (40%) | 559 (43%) |  |
| Below average (80-89) | 16 (10%) | 34 (6%) | 13 (1%) |  |
| Borderline impairment (70-79) | 7 (4%) | 23 (4%) | 12 (1%) |  |
| Mild impairment (55-69) | 14 (8%) | 12 (2%) | 0 (0%) |  |
| Moderate impairment (40-54) | 17 (10%) | 7 (1%) | 0 (0%) |  |
| Severe impairment (21 to 39) | 4 (2%) | 1 (<1%) | 0 (0%) |  |
| Profound impairment (<20) | 1 (1%) | 1 (<1%) | 0 (0%) |  |
| **Non-ASD Diagnoses (n, %)** |  |  |  |  |
| ID/GDD | 17 (10%) | 23 (4%) | - | 8.2 (.004) |
| Speech/language disorder | 31 (18%) | 98 (18%) | - | <0.1 (.946) |
| ADHD | 53 (31%) | 262 (48%) | - | 15.2 (<.001) |
| ODD/CD | 13 (8%) | 46 (9%) | - | 0.1 (.739) |
| Anxiety disorder | 36 (21%) | 192 (36%) | - | 11.9 (<.001) |
| Specific learning disorder | 9 (5%) | 62 (12%) | - | 5.4 (.020) |
| Motor / coordination disorder | 9 (5%) | 19 (4%) | - | 1.1 (.290) |
| Depressive disorder | 17 (10%) | 83 (15%) | - | 3.0 (.085) |
| Bipolar disorder / mania | 4 (2%) | 7 (1%) | - | 1.0 (.324) |
| Obsessive compulsive disorder | 10 (6%) | 16 (3%) | - | 3.2 (.074) |
| Tic disorder | 4 (2%) | 5 (1%) | - | 2.1 (.143) |
| Feeding / eating disorder | 7 (4%) | 21 (4%) | - | <0.1 (.879) |

Note. NT=neurotypical controls, DD=non-ASD developmental disability, ASD=autism spectrum disorder. ID/GDD=Intellectual disability/global developmental delay, ADHD=Attention-Deficit/Hyperactivity disorder; ODD/CD=oppositional defiant disorder/conduct disorder. Non-ASD diagnoses do not sum to 100% because children could be diagnosed with more than one condition.

**Online Supplement 5.** Descriptive statistics for all variables included in structural models.

|  | **Mean** | **SD** | **Min** | **Max** |
| --- | --- | --- | --- | --- |
| 1. SSDS total | 244.7 | 36.4 | 96.0 | 309.0 |
| 2. Social Motivation | 55.1 | 10.7 | 14.0 | 70.0 |
| 3. Social Affiliation | 28.6 | 5.1 | 7.0 | 35.0 |
| 4. Social Communication | 32.7 | 5.4 | 9.0 | 40.0 |
| 5. Social Recognition | 27.1 | 5.2 | 7.0 | 35.0 |
| 6. Unusual Social Approach | 6.4 | 3.0 | 1.0 | 17.0 |
| 7. EFS total | 3.5 | 0.6 | 1.6 | 5.0 |
| 8. Sequencing / Working Memory | 3.9 | 0.8 | 1.0 | 5.0 |
| 9. Risk Avoidance | 3.4 | 0.8 | 1.0 | 5.0 |
| 10. Response Inhibition | 3.2 | 0.7 | 1.0 | 5.0 |
| 11. Emotion Regulation | 3.4 | 0.8 | 1.0 | 5.0 |
| 12. Set Shifting | 3.6 | 0.7 | 1.0 | 5.0 |
| 13. OS-CBS total | 1.4 | 0.5 | 1.0 | 5.0 |
| 14. Property Destruction | 1.5 | 0.7 | 1.0 | 5.0 |
| 15. Aggression | 1.3 | 0.5 | 1.0 | 5.0 |
| 16. Elopement | 1.3 | 0.6 | 1.0 | 5.0 |
| 17. Conduct Problems | 1.7 | 0.7 | 1.0 | 5.0 |
| 18. Self-Injury | 1.2 | 0.4 | 1.0 | 5.0 |
| 19. CFQL-2 total | 3.7 | 0.6 | 1.4 | 5.0 |
| 20. Child | 3.5 | 0.5 | 1.5 | 5.0 |
| 21. Family | 3.7 | 0.9 | 1.0 | 5.0 |
| 22. Caregiver | 3.6 | 0.8 | 1.0 | 5.0 |
| 23. Financial | 3.9 | 0.7 | 1.0 | 5.0 |
| 24. External support | 3.8 | 0.7 | 1.0 | 5.0 |
| 25. Partner relationship | 4.1 | 0.8 | 1.0 | 5.0 |
| 26. Coping | 3.8 | 0.7 | 1.0 | 5.0 |
| 27. Parent and child impact | 3.1 | 0.4 | 1.0 | 5.0 |
| 28. Medical and legal impact | 0.0 | 0.7 | -0.4 | 3.9 |

**Online Supplement 6.** Bivariate correlations between all variables included in structural models.

|  | **1** | **2** | **3** | **4** | **5** | **6** | **7** | **8** | **9** | **10** | **11** | **12** | **13** | **14** | **15** | **16** |
| --- | --- | --- | --- | --- | --- | --- | --- | --- | --- | --- | --- | --- | --- | --- | --- | --- |
| 1. SSDS total | - | .88 | .78 | .90 | .77 | -.58 | .60 | .56 | .23 | .48 | .45 | .60 | -.37 | -.29 | -.28 | -.34 |
| 2. Social Motivation |  | - | .64 | .74 | .50 | -.48 | .46 | .38 | .11 | .31 | .42 | .49 | -.28 | -.21 | -.21 | -.22 |
| 3. Social Affiliation |  |  | - | .70 | .51 | -.29 | .36 | .37 | .07 | .30 | .24 | .38 | -.17 | -.11 | -.12 | -.20 |
| 4. Social Communication |  |  |  | - | .68 | -.42 | .52 | .49 | .17 | .41 | .40 | .52 | -.31 | -.25 | -.25 | -.27 |
| 5. Social Recognition |  |  |  |  | - | -.46 | .63 | .63 | .33 | .58 | .40 | .58 | -.42 | -.34 | -.30 | -.39 |
| 6. Unusual Social Approach |  |  |  |  |  | - | -.53 | -.41 | -.34 | -.40 | -.44 | -.51 | .42 | .36 | .31 | .30 |
| 7. EFS total |  |  |  |  |  |  | - | .85 | .61 | .85 | .82 | .92 | -.66 | -.57 | -.49 | -.45 |
| 8. Sequencing / Working Memory |  |  |  |  |  |  |  | - | .40 | .69 | .52 | .76 | -.51 | -.43 | -.35 | -.41 |
| 9. Risk Avoidance |  |  |  |  |  |  |  |  | - | .52 | .44 | .44 | -.54 | -.48 | -.39 | -.45 |
| 10. Response Inhibition |  |  |  |  |  |  |  |  |  | - | .60 | .71 | -.58 | -.52 | -.43 | -.38 |
| 11. Emotion Regulation |  |  |  |  |  |  |  |  |  |  | - | .71 | -.57 | -.50 | -.44 | -.28 |
| 12. Set Shifting |  |  |  |  |  |  |  |  |  |  |  | - | -.55 | -.47 | -.41 | -.37 |
| 13. OS-CBS total |  |  |  |  |  |  |  |  |  |  |  |  | - | .88 | .86 | .68 |
| 14. Property Destruction |  |  |  |  |  |  |  |  |  |  |  |  |  | - | .76 | .47 |
| 15. Aggression |  |  |  |  |  |  |  |  |  |  |  |  |  |  | - | .45 |
| 16. Elopement |  |  |  |  |  |  |  |  |  |  |  |  |  |  |  | - |
| 17. Conduct Problems |  |  |  |  |  |  |  |  |  |  |  |  |  |  |  |  |
| 18. Self-Injury |  |  |  |  |  |  |  |  |  |  |  |  |  |  |  |  |
| 19. CFQL-2 total |  |  |  |  |  |  |  |  |  |  |  |  |  |  |  |  |
| 20. Child |  |  |  |  |  |  |  |  |  |  |  |  |  |  |  |  |
| 21. Family |  |  |  |  |  |  |  |  |  |  |  |  |  |  |  |  |
| 22. Caregiver |  |  |  |  |  |  |  |  |  |  |  |  |  |  |  |  |
| 23. Financial |  |  |  |  |  |  |  |  |  |  |  |  |  |  |  |  |
| 24. External support |  |  |  |  |  |  |  |  |  |  |  |  |  |  |  |  |
| 25. Partner relationship |  |  |  |  |  |  |  |  |  |  |  |  |  |  |  |  |
| 26. Coping |  |  |  |  |  |  |  |  |  |  |  |  |  |  |  |  |
| 27. Parent and child impact |  |  |  |  |  |  |  |  |  |  |  |  |  |  |  |  |
| 28. Medical and legal impact |  |  |  |  |  |  |  |  |  |  |  |  |  |  |  |  |

|  | **17** | **18** | **19** | **20** | **21** | **22** | **23** | **24** | **25** | **26** | **27** | **28** |
| --- | --- | --- | --- | --- | --- | --- | --- | --- | --- | --- | --- | --- |
| 1. SSDS total | -.33 | -.28 | .47 | .41 | .45 | .33 | .41 | .33 | .23 | .33 | -.41 | -.13 |
| 2. Social Motivation | -.24 | -.27 | .44 | .40 | .41 | .28 | .38 | .32 | .22 | .33 | -.40 | -.13 |
| 3. Social Affiliation | -.12 | -.17 | .31 | .28 | .29 | .19 | .30 | .20 | .16 | .21 | -.29 | -.11 |
| 4. Social Communication | -.26 | -.25 | .42 | .39 | .39 | .28 | .38 | .30 | .22 | .30 | -.38 | -.13 |
| 5. Social Recognition | -.39 | -.22 | .39 | .32 | .37 | .32 | .33 | .28 | .17 | .28 | -.32 | -.08 |
| 6. Unusual Social Approach | .39 | .27 | -.41 | -.35 | -.40 | -.34 | -.34 | -.28 | -.14 | -.31 | .35 | .12 |
| 7. EFS total | -.67 | -.37 | .72 | .64 | .66 | .64 | .46 | .46 | .33 | .57 | -.49 | -.14 |
| 8. Sequencing / Working Memory | -.51 | -.27 | .54 | .47 | .50 | .48 | .37 | .36 | .26 | .40 | -.34 | -.08 |
| 9. Risk Avoidance | -.53 | -.25 | .40 | .29 | .38 | .41 | .25 | .24 | .17 | .34 | -.32 | -.12 |
| 10. Response Inhibition | -.62 | -.26 | .56 | .47 | .52 | .56 | .33 | .34 | .28 | .44 | -.35 | -.10 |
| 11. Emotion Regulation | -.59 | -.36 | .71 | .67 | .67 | .62 | .42 | .45 | .32 | .60 | -.50 | -.15 |
| 12. Set Shifting | -.56 | -.36 | .65 | .60 | .60 | .55 | .45 | .42 | .30 | .51 | -.47 | -.14 |
| 13. OS-CBS total | .90 | .63 | -.58 | -.44 | -.58 | -.55 | -.39 | -.34 | -.23 | -.49 | .55 | .22 |
| 14. Property Destruction | .76 | .45 | -.51 | -.39 | -.51 | -.51 | -.32 | -.27 | -.20 | -.44 | .44 | .16 |
| 15. Aggression | .71 | .44 | -.47 | -.34 | -.47 | -.46 | -.32 | -.24 | -.19 | -.42 | .46 | .19 |
| 16. Elopement | .51 | .39 | -.32 | -.20 | -.33 | -.28 | -.25 | -.26 | -.11 | -.23 | .38 | .17 |
| 17. Conduct Problems | - | .44 | -.58 | -.47 | -.59 | -.57 | -.35 | -.32 | -.25 | -.51 | .49 | .16 |
| 18. Self-Injury |  | - | -.38 | -.30 | -.36 | -.29 | -.30 | -.26 | -.14 | -.32 | .43 | .25 |
| 19. CFQL-2 total |  |  | - | .75 | .88 | .84 | .69 | .71 | .59 | .82 | -.59 | -.19 |
| 20. Child |  |  |  | - | .66 | .59 | .39 | .42 | .34 | .59 | -.47 | -.15 |
| 21. Family |  |  |  |  | - | .75 | .57 | .49 | .36 | .73 | -.59 | -.18 |
| 22. Caregiver |  |  |  |  |  | - | .47 | .48 | .36 | .71 | -.43 | -.11 |
| 23. Financial |  |  |  |  |  |  | - | .46 | .30 | .49 | -.52 | -.22 |
| 24. External support |  |  |  |  |  |  |  | - | .41 | .47 | -.37 | -.11 |
| 25. Partner relationship |  |  |  |  |  |  |  |  | - | .35 | -.20 | -.05 |
| 26. Coping |  |  |  |  |  |  |  |  |  | - | -.50 | -.16 |
| 27. Parent and child impact |  |  |  |  |  |  |  |  |  |  | - | .31 |
| 28. Medical and legal impact |  |  |  |  |  |  |  |  |  |  |  | - |

**Note. All bivariate correlations are statistically significant p<.05**

**Online Supplement 7.** Standardized multi-group structural model estimates for each group, absolute differences in estimates, and summary statistics for Aim 1.

|  | **Diagnostic Groups** | | | **Sex** | | | **Age** | | |
| --- | --- | --- | --- | --- | --- | --- | --- | --- | --- |
|  | NT | DD | \|Δ\| | Female | Male | \|Δ\| | <8 | ≥8 | \|Δ\| |
| Social Motivation | -.06 | .03 | .08 | -.04 | -.03 | .01 | -.03 | -.04 | .01 |
| Social Affiliation | .08 | .05 | .03 | .10 | .05 | .05 | .09 | .04 | .05 |
| Social Communication | .08 | -.08 | .16 | -.03 | .00 | .04 | -.05 | -.01 | .03 |
| Social Recognition | -.05 | -.01 | .04 | -.03 | -.02 | .01 | -.02 | -.02 | .00 |
| Unusual Social Approach | .06 | .15 | .09 | .06 | .12 | .06 | .05 | .12 | .07 |
| Sequencing / Working Memory | -.17 | -.04 | .13 | -.10 | -.10 | .01 | -.08 | -.08 | .00 |
| Risk Avoidance | -.21 | -.30 | .09 | -.26 | -.25 | .01 | -.28 | -.23 | .05 |
| Response Inhibition | -.18 | -.21 | .03 | -.20 | -.18 | .02 | -.18 | -.16 | .03 |
| Emotion Regulation | -.23 | -.17 | .05 | -.23 | -.23 | .01 | -.29 | -.22 | .08 |
| Set Shifting | .01 | -.03 | .04 | -.03 | .00 | .03 | -.01 | -.03 | .02 |
|  | Average \|Δ\| | | .07 | Average \|Δ\| | | .02 | Average \|Δ\| | | .03 |
|  | r | | .76 | r | | .97 | r | | .96 |
| Constrained Model Fit | Χ^2^(10)=71.9, p<.001  RMSEA=.079  CFI=.947  TLI=.894 | | | Χ^2^(10)=23.8, p=.008  RMSEA=.037  CFI=.990  TLI=.979 | | | Χ^2^(10)=19.0, p=.040  RMSEA=.030  CFI=.993  TLI=.986 | | |
|  |  |  |  |  |  |  |  |  |  |

Note. NT=neurotypical. DD=developmental disability. Unconstrained models were just identified.

**Online Supplement 8.** Standardized multi-group structural model estimates for each group, absolute differences in estimates, and summary statistics for Aim 2.

|  | **Diagnostic Groups** | | | **Sex** | | | **Age** | | |
| --- | --- | --- | --- | --- | --- | --- | --- | --- | --- |
|  | NT | DD | \|Δ\| | Female | Male | \|Δ\| | <8 | ≥8 | \|Δ\| |
| Outcome: CFQL-2 Total Score | | | | | | | | | |
| Property Destruction | -.12 | -.12 | .00 | -.07 | -.15 | .07 | -.03 | -.22 | .19 |
| Aggression | -.12 | .02 | .14 | -.10 | .02 | .13 | -.10 | .06 | .16 |
| Elopement | .04 | <.00 | .04 | .05 | -.03 | .08 | -.08 | .02 | .11 |
| Conduct Problems | -.37 | -.42 | .06 | -.39 | -.46 | .07 | -.45 | -.44 | .01 |
| Self-Injury | .02 | -.15 | .17 | -.18 | -.07 | .11 | -.08 | -.12 | .04 |
| Outcome: Parent / Child Impact | | | | | | | | | |
| Property Destruction | -.04 | .06 | .10 | <.01 | .02 | .01 | -.04 | .13 | .17 |
| Aggression | .18 | .16 | .02 | .13 | .13 | .00 | .14 | .06 | .08 |
| Elopement | .08 | .14 | .06 | .02 | .19 | .17 | .28 | .15 | .13 |
| Conduct Problems | .17 | .19 | .01 | .18 | .26 | .08 | .15 | .31 | .17 |
| Self-Injury | .01 | .20 | .19 | .31 | .13 | .18 | .21 | .13 | .08 |
| Outcome: Medical / Legal Impact | | | | | | | | | |
| Property Destruction | -.06 | -.03 | .04 | .00 | -.07 | .07 | -.10 | <.01 | .10 |
| Aggression | .04 | .16 | .12 | .15 | .08 | .07 | .10 | .08 | .01 |
| Elopement | .07 | .05 | .02 | .04 | .07 | .03 | .08 | .09 | .01 |
| Conduct Problems | <.01 | -.01 | .01 | -.02 | .02 | .04 | -.01 | .02 | .03 |
| Self-Injury | .14 | .21 | .07 | .25 | .17 | .08 | .22 | .16 | .06 |
|  | Average \|Δ\| | | .07 | Average \|Δ\| | | .08 | Average \|Δ\| | | .09 |
|  | r | | .85 | r | | .84 | r | | .81 |
| Constrained Model Fit | Χ^2^(15)=140.1, p<.001  RMSEA=.091  CFI=.917  TLI=.801 | | | Χ^2^(15)=57.8, p<.001  RMSEA=.053  CFI=.981  TLI=.955 | | | Χ^2^(15)=147.3, p<.001  RMSEA=.094  CFI=.946  TLI=.871 | | |
|  |  |  |  |  |  |  |  |  |  |

Note. NT=neurotypical. DD=developmental disability. Unconstrained models were just identified.

**Online Supplement 9.** Standardized multi-group structural model estimates for each group, absolute differences in estimates, and summary statistics for Aim 3.

|  | **Diagnostic Groups** | | | **Sex** | | | **Age** | | |
| --- | --- | --- | --- | --- | --- | --- | --- | --- | --- |
|  | NT | DD | \|Δ\| | Female | Male | \|Δ\| | <8 | ≥8 | \|Δ\| |
| SSDS-CFQL-2: Indirect | .01 | <.01 | .01 | <.01 | .02 | .01 | .01 | .03 | .02 |
| SSDS-CFQL-2: Direct | .19 | .17 | .02 | .26 | .23 | .03 | .13 | .20 | .07 |
| EFS-CFQL-2: Indirect | .07 | .08 | .01 | .08 | .10 | .01 | .09 | .10 | .00 |
| EFS-CFQL-2: Direct | .28 | .25 | .04 | .30 | .27 | .02 | .29 | .38 | .09 |
| SSDS-P/C Impact: Indirect | -.01 | -.01 | .00 | -.01 | -.03 | .02 | -.01 | -.05 | .04 |
| SSDS-P/C Impact: Direct | -.18 | -.23 | .05 | -.33 | -.28 | .06 | -.25 | -.23 | .01 |
| EFS-P/C Impact: Indirect | -.08 | -.14 | .06 | -.10 | -.15 | .05 | -.10 | -.17 | .06 |
| EFS-P/C Impact: Direct | -.11 | -.03 | .08 | -.10 | -.06 | .05 | -.08 | -.15 | .07 |
| SSDS-M/L Impact: Indirect | <.01 | <.01 | .00 | <.01 | -.01 | .01 | <.01 | -.02 | .02 |
| SSDS-M/L Impact: Direct | -.05 | -.10 | .05 | -.11 | -.11 | .01 | -.11 | -.07 | .04 |
| EFS-M/L Impact: Indirect | -.02 | -.06 | .05 | -.07 | -.05 | .02 | -.03 | -.07 | .05 |
| EFS-M/L Impact: Direct | -.01 | .05 | .06 | -.03 | .06 | .09 | .01 | <.01 | .02 |
|  | Average \|Δ\| | | .04 | Average \|Δ\| | | .03 | Average \|Δ\| | | .04 |
|  | r | | .94 | r | | .97 | R | | .97 |
| Constrained Model Fit | Χ^2^(11)=110.8, p<.001  RMSEA=.095  CFI=.932  TLI=.827 | | | Χ^2^(11)=33.0, p=.001  RMSEA=.045  CFI=.990  TLI=.974 | | | Χ^2^(11)=142.1, p<.001  RMSEA=.102  CFI=.944  TLI=.857 | | |
|  |  |  |  |  |  |  |  |  |  |

Note. NT=neurotypical. DD=developmental disability. P/C Impact = Parent / Child Impact. M/L Impact = Medical / Legal Impact. Unconstrained models were just identified.
